# Large‐Area Bright Emission of Plasmon‐Coupled Dark Excitons at Room Temperature

**DOI:** 10.1002/advs.202411841

**Published:** 2024-11-28

**Authors:** Hyun Jeong, Hyeong Chan Suh, Ga Hyun Cho, Huitae Joo, Yeonjeong Koo, Hayoung Ko, Ki Kang Kim, Youngbum Kim, Jeongyong Kim, Kyoung‐Duck Park, Mun Seok Jeong

**Affiliations:** ^1^ Department of Physics Hanyang University Seoul 04763 Republic of Korea; ^2^ Department of Physics Pohang University of Science and Technology (POSTECH) Pohang 37673 Republic of Korea; ^3^ Department of Energy Science Sungkyunkwan University Suwon 16419 Republic of Korea; ^4^ Center for Integrated Nanostructure Physics (CINAP) Institute for Basic Science (IBS) Sungkyunkwan University Suwon 16419 Republic of Korea

**Keywords:** dark exciton, monolayer WSe_2_, photoluminescence, strain, surface plasmon

## Abstract

Brightening dark excitons in transition metal dichalcogenide monolayers (MLs) can provide large‐area ultrathin devices for applications in quantum information science and optoelectronics. For practical applications of dark excitons, a robust and bright emission over a wide area at room temperature is desirable; however, no reliable approach has been demonstrated thus far. In this study, an efficient approach is presented for brightening dark excitons at room temperature over a large area of a WSe_2_ ML via coupling between plasmons and dark excitons. When a WSe_2_ ML is placed on gold micropillars (Au MPs), dark excitons are efficiently coupled to strongly localized surface plasmons at the edges of the Au MPs, along with a strong photoluminescence (PL) emission. Room‐temperature dark exciton emission is confirmed via energy‐, angle‐, and time‐resolved spectroscopy experiments, as well as confocal PL mapping. This study provides a generalizable method for the practical application of dark exciton.

## Graphical abstract

A bright emission of dark excitons in WSe_2_ over a large area at room temperature is reported. By transferring a WSe_2_ monolayer onto the Au micropillars, dark excitons are funneled and coupled to the Au surface plasmons to enhance the emission. This enables the possibility of practically using of dark excitons in optoelectronic applications, such as light‐emitting devices and solar cells.

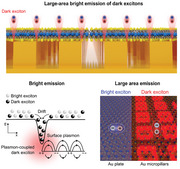

## 1 Introduction

Transition metal dichalcogenide (TMD) monolayers (MLs) such as MoS_2_ and WSe_2_ are direct‐bandgap semiconducting materials that are promising building blocks for optoelectronic applications.^[^ ^1^, ^2^, ^3^, ^4^, ^5^, ^6^ ^]^ The strong spin‐orbit coupling of TMD MLs is an intriguing feature.^[^ ^7^, ^8^, ^9^, ^10^ ^]^ Spin‐orbit coupling in TMD MLs can lead to the generation of two types of excitons^[^ ^11^, ^12^, ^13^ ^]^: bright and dark, which are composed of electrons and holes and are distinguished according to the electron spin direction in the energy state of the split conduction band.^[^ ^14^, ^15^, ^16^ ^]^ The lifetimes of the bright and dark excitons are <10 and >100 ps, respectively, demonstrating a significant difference.^[^ ^17^, ^18^, ^19^ ^]^ Dark excitonic states may accumulate into an enormous population as their energy levels are lower than that of the bright excitonic state.^[^ ^20^, ^21^ ^]^ In addition, owing to the unique characteristics of the out‐of‐plane dipole orientation,^[^ ^22^, ^23^ ^]^ dark excitonic states in TMD MLs are more promising compared to the bright excitonic states for condensation, valleytronic, and exciton transport applications.^[^ ^24^, ^25^, ^26^, ^27^ ^]^ Given these advantages, recent studies have investigated the properties and emission of dark excitons. Zhang et al. demonstrated that in‐plane magnetic fields greater than 10 T under cryogenic temperatures enable the brightening of dark excitons in a WSe_2_ ML.^[^ ^12^ ^]^ Zhou et al. probed dark excitons in a WSe_2_ ML via near‐field coupling to surface plasmon polaritons at a low temperature of ≈4 K.^[^ ^23^ ^]^ Using a plasmonic tip, Park et al. enabled the radiative control of dark excitons in a WSe_2_ ML at room temperature.^[^ ^15^ ^]^ In addition, Gelly et al. proposed probing dark exciton navigation via the local‐strain landscape of a WSe_2_ ML.^[^ ^28^ ^]^ However, the observations of dark excitons reported thus far have limitations as they require an external magnetic field, are conducted at low temperatures, or are restricted to localized areas. In particular, the large‐area emission of dark excitons is required for practical applications in light‐emitting devices, spintronics, photovoltaics, and excitonic devices.^[^ ^16^, ^29^, ^30^, ^31^ ^]^

In this study, we observed dark exciton emissions in a WSe_2_ ML over a large area at room temperature with no external magnetic fields by placing the 2D crystal on periodic gold micropillars (Au MPs). The surface properties of the fabricated WSe_2_ ML on Au MPs were investigated using atomic force microscopy (AFM) and scanning electron microscopy (SEM), and the radiative emission properties were investigated using photoluminescence (PL) spectroscopy and time‐resolved PL, while also characterizing the surface plasmon (SP) resonance. Finite‐difference time‐domain (FDTD) simulations were conducted to investigate the PL enhancement mechanism of dark excitons coupled with Au MPs. In addition, confocal PL mapping experiments of the device for bright and dark excitons enabled a better understanding of the dark exciton emission mechanism over a large 2D area attributed to the local strain effect. Angle‐resolved PL spectroscopy revealed distinct signatures of dark exciton emission induced by the out‐of‐plane transition dipole moment.

## 2 Results and Discussion

### 2.1 Fabrication of the WSe_2_ ML on Large‐Scale Periodic Au MPs

**Figure 1:**
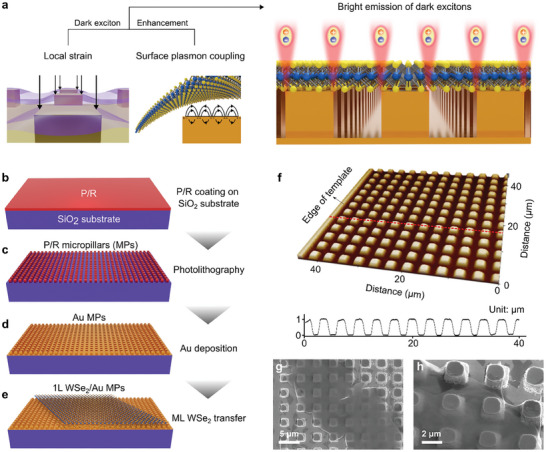
Fabrication process and surface morphologies of the WSe_2_ ML on Au MPs. a) 3D schematic of large‐area dark exciton emission from the WSe_2_ ML on Au MPs, facilitated by the high local strain and coupling with the surface plasmon. 3D illustrations of the fabrication process, including the b) P/R coating on the SiO_2_ substrate, c) conventional photolithography process to form the P/R MPs, d) 100‐nm‐thick Au deposition using sputtering to produce the Au MPs, and e) transfer of the CVD‐grown WSe_2_ ML onto the Au MPs using the wet transfer method. f) AFM topography of the Au MPs array and height profiles along the cross‐sectional line, as indicated by the red dotted line. The diameter, height, and periodicity of the Au MPs were 2, 1, and 4 µm, respectively. SEM image of the WSe_2_ ML on the Au MPs with g) low and h) high magnifications.

**Figure** **1**a presents a 3D schematic of the large‐area dark exciton emission in the WSe_2_ ML. A local strain was induced in the WSe_2_ ML at the edges of the Au MP, resulting in the emission of dark excitons.^[^ ^28^ ^]^ In addition, the coupling between the surface plasmons and out‐of‐plane dark excitons enhanced the emission of dark excitons.^[^ ^32^ ^]^ Consequently, the bright emission of the dark excitons was facilitated over a large area of the 2D semiconductor, even at room temperature. A square array of periodic Au MPs was used as a substrate for transferring the WSe_2_ ML. The Au MP array was fabricated using conventional photolithography, which is inexpensive, requires less time, and allows large‐area processing. The Au MP arrays were fabricated as follows. i) A photoresist (P/R) was coated onto a SiO_2_ substrate (Figure 1b). ii) Periodic P/R MPs were formed using conventional photolithography (Figure 1c). iii) A 100‐nm‐thick Au film was deposited on the P/R MP array via RF sputtering to form the Au MPs (Figure 1d). The Au MP array formed over a large area at a scale greater than one hundred micrometers (Figure S1, Supporting Information). The individual Au MPs were uniform in size and arranged in a square array with regular spacing between them (Figure S2, Supporting Information). The WSe_2_ ML grown via chemical vapor deposition (CVD) were transferred onto the Au MPs using a wet transfer method, as depicted in Figure 1e. The wet transfer of the WSe_2_ ML was performed using a polymethyl methacrylate (PMMA)‐assisted method (Figure S3, Supporting Information). The WSe_2_ ML used in this study was triangular and was confirmed to be a monolayer via AFM topography. (Figure S4, Supporting Information). The WSe_2_ ML on the SiO_2_ substrate was 0.7 nm thick, which is consistent with the thickness of typical WSe_2_ MLs.^[^ ^33^, ^34^, ^35^ ^]^ The WSe_2_ ML was successfully transferred to the Au MPs owing to its high in‐plane stiffness, which was attributed to the strong covalent bonds between the transition metal and chalcogen atoms.^[^ ^36^ ^]^ The top image in Figure 1f is the 3D AFM topography of the Au MP array. The size of the Au MP was considerably uniform, and the array was almost perfectly periodic. The straight and flat area on the left edge of the AFM topography represents the end of the Au MP template. The bottom plot in Figure 1f demonstrates the height profiles along the cross‐section, as indicated by the red dotted line in the AFM topography. The diameter, height, and periodicity of the Au MPs were 2, 1, and 4 µm, respectively. To characterize the surface morphology of the WSe_2_ ML transferred onto the Au MPs, top‐view scanning electron microscopy (SEM) images were obtained at low (Figure 1g) and high magnifications (Figure 1h). The WSe_2_ ML transferred to the Au MPs covered several tens of Au MPs and was under strong stress at the edges of the Au MPs. This strong local stress was a natural consequence of the wet transfer of the WSe_2_ ML onto the Au MPs. Note that at the edge of the Au MP, the WSe_2_ ML was under a tensile strain in all directions, and this local strain was sufficiently large to modify the crystal structure (Figure S5, Supporting Information). This strain can generate an energy potential in a localized region that funnels nearby excitons. Therefore, dark excitons with lifetimes longer than bright excitons can reach the energetic minimum to be observed under these conditions.^[^ ^28^ ^]^ The strain of WSe_2_ ML in the localized region is 0.70%–1.17%. (Figure S6, Supporting Information) This range allows the funneling of excitons in the WSe_2_ ML, while at the same time being the range where the strain‐induced indirect transition is not dominant.^[^ ^37^, ^38^ ^]^

### 2.2 Optical Properties of the WSe_2_ ML on Au MPs

**Figure 2:**
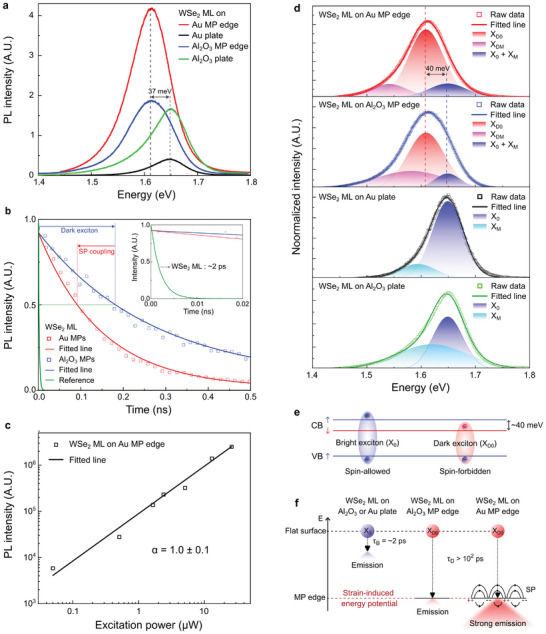
Optical properties of the WSe_2_ ML on the Au MP template. a) Room‐temperature PL spectra of the WSe_2_ ML on the Au MP edge, Au plate, Al_2_O_3_ MP edge, and Al_2_O_3_ plate. b) Time‐resolved photoluminescence curves of the WSe_2_ ML on the Al_2_O_3_ and Au MP edges. c) Excitation power‐dependent PL intensity of the dark excitons in a log plot. Fitting of the dark exciton emissions with a linear power dependence is represented by the black line. d) Intensity‐normalized PL spectra of the WSe_2_ ML on various templates with embedded exciton and a combination of multiple excitons peaks fitted by the Gaussian model. e) Split‐band configuration of the bright and dark exciton states according to the spin direction of the electron. f) Illustration of the difference in the drift of the bright and dark excitons between the flat surface and MP edge.

Because the WSe_2_ ML transferred to the Au MP is naturally under highly stressed conditions, changes in its quantum physical properties are expected, such as excitonic absorption, charge separation, and variation in the energy bandgap.^[^ ^39^, ^40^, ^41^ ^]^ Therefore, optical characterizations of the WSe_2_ ML on Au MPs using spectral and temporal PL spectroscopy were performed. **Figure** **2**a presents the PL spectra of the WSe_2_ ML on the Au MP edge, Au plate, Al_2_O_3_ MP edge, and Al_2_O_3_ plate, indicated by red, black, blue, and green solid lines, respectively. The Au plate and Al_2_O_3_ MP edge were used as references to compare the strain and SP coupling effects of the WSe_2_ ML on the Au MP edge, respectively. An Al_2_O_3_ plate was used as a reference to verify the PL quenching effect of Au. The peak energies of the PL spectra of the WSe_2_ ML on the Au and Al_2_O_3_ MPs were consistent at 1.612 eV, which was red‐shifted by ≈37 meV compared to the peak energy of the PL spectrum of the WSe_2_ ML on the Au and Al_2_O_3_ plates at 1.649 eV. The peak energy difference of ≈37 meV is in agreement with the energy difference between the bright and dark excitons of the WSe_2_ ML, as previously reported.^[^ ^12^, ^15^, ^23^, ^42^, ^43^ ^]^ This supports the observation of dark excitons in the WSe_2_ ML at room temperature owing to the large strain at the MP edge, regardless of substrate material. However, the PL intensity of the WSe_2_ ML exhibits a notable behavior depending on the substrate. For the Al_2_O_3_ substrates, the PL intensity of the WSe_2_ ML was not significantly different between the MP edge and plate. Compared with the Al_2_O_3_ plate, the integrated PL intensity of the WSe_2_ ML on the Al_2_O_3_ MP edge increased by ≈25%, and the full width at half maximum (FWHM) increased by ≈18 meV. This is because the large strain of the WSe_2_ ML at the MP edge only brightened the dark excitons. Conversely, for the Au substrate, the PL intensity of the WSe_2_ ML dramatically varied between the MP edge and plate. The integrated PL intensity of the WSe_2_ ML at the Au MP edge increased by ≈1169%, and the FWHM increased by ≈18 meV compared to the Au plate. This occurred owing to the coupling with the Au SP, which enhanced the dark exciton emission of the WSe_2_ ML that was brightened at the Au MP edge. Note that the PL intensity of the WSe_2_ ML on the Au plate was smaller than that of the WSe_2_ ML on the Al_2_O_3_ plate owing to PL quenching by charge transfer.^[^ ^44^ ^]^ These results indicate that the PL enhancement of the WSe_2_ ML on the Au MP edge was dominated by surface plasmon resonance, whereas the PL of the WSe_2_ ML on the Au plate was dominated by PL quenching owing to charge transfer. In WSe_2_ MLs, dark excitons typically have longer lifetimes than bright excitons because they require a spin flip to recombine, which does not occur via long‐range electron‐hole exchange interactions. ^[^ ^20^, ^23^, ^45^, ^46^, ^47^, ^48^ ^]^ The lifetimes of bright and dark excitons in WSe2 ML are reported to be ≈2 and >100 ps, respectively, demonstrating a significant difference of two orders of magnitude even at room temperature.^[^ ^14^, ^17^, ^46^, ^49^, ^50^ ^]^ The PL decay curves of the WSe_2_ ML on Al_2_O_3_ and Au MPs are shown in Figure 2b. The red and blue hollowed squares represent the measured values of the WSe_2_ ML on the Au MPs and Al_2_O_3_ MPs, respectively. The solid lines represent the PL decay curves fitted using a single‐exponential decay model. The exciton lifetimes of the WSe_2_ ML on the Au and Al_2_O_3_ MPs obtained by fitting were 161 and 305 ps, respectively. Both values were longer than 100 ps, which corresponds to the lifetime of dark excitons.^[^ ^46^ ^]^ These results demonstrate that dark excitons in the WSe_2_ ML on MPs can be observed owing to the local strain induced by the sharp MP edge. The possibility of bright trion emission can be excluded because the lifetime of bright trions in the WSe_2_ ML is ≈10 ps.^[^ ^21^ ^]^ Note that the exciton lifetime of the WSe_2_ ML on Au MPs was ≈47% shorter than that of the WSe_2_ ML on Al_2_O_3_ MPs. The PL lifetime decreases because the coupling between the exciton and SP increases the radiative decay rate.^[^ ^51^, ^52^ ^]^ This increased the spontaneous emission rate, resulting in an increase in the PL intensity with Purcell enhancement.^[^ ^53^, ^54^, ^55^ ^]^ It indicates a coupling between the dark exciton of the WSe_2_ ML and the SP of Au at the MP edge. Note that the lifetime of the WSe_2_ ML on the Au plate, corresponding to a bright exciton, was observed to be 104 ± 3 ps, as the temporal resolution of the time‐resolved PL used in this study was limited to 100 ps (Figure S7, Supporting Information). Note that less than 14% of the bright‐state component was still present in the TRPL measurement of the dark exciton PL. We measured the power‐dependent PL intensity of dark excitons to determine the possible contribution of bi‐excitons, which have comparable photon energy to that of dark excitons. Figure 2c presents a plot of the PL intensity as a function of the excitation power of the dark excitons in the WSe_2_ ML on the Au MPs. The black hollow squares and solid lines represent the raw data and curve fit of the dark exciton, respectively. For the dark excitons, we found that the integrated PL intensity linearly increased as the excitation power increased, excluding a bi‐exciton contribution.^[^ ^15^, ^56^ ^]^ An excitonic peak analysis was performed to analyze further the changes in the optical properties of the WSe_2_ MLs on each substrate. Figure 2d presents the intensity‐normalized PL spectra of the WSe_2_ ML on the Au MP edge, Al_2_O_3_ MP edge, Au plate, and Al_2_O_3_ plate substrates with excitonic peaks, ordered from top to bottom. The raw data and fitted PL spectra are represented by hollow squares and solid lines, respectively. An area‐normalized Gaussian function was used to analyze the excitonic peaks in the PL spectrum.^[^ ^57^, ^58^ ^]^ X_0_, X_M_, X_D0_, and X_DM_ denote bright excitons, a combination of bright multiple excitons, dark excitons, and a combination of dark multiple excitons, respectively. In Figure 2d, the embedded excitonic peaks extracted by Gaussian fitting are colored red, magenta, and blue, which represent the dark excitons, combination of dark multiple excitons, and bright excitons, respectively, including the combination of bright multiple excitons in the PL spectra of the WSe_2_ ML on the Au and Al_2_O_3_ MP edges. The bright excitons and combination of bright multiple excitons embedded in the PL spectra of the WSe_2_ ML on the Au and Al_2_O_3_ plates are shown in blue and cyan, respectively. The peak energies of the dark excitons and the combination of dark multiple excitons of the WSe_2_ ML at the Au and Al_2_O_3_ MPs edges were 1.608 and 1.574 eV, respectively. The combined bright exciton energy of the WSe_2_ ML on the Au and Al_2_O_3_ MP edges was 1.648 eV, which is nearly identical to the PL peak energy of the WSe_2_ ML on the Au and Al_2_O_3_ plates, as shown in Figure 2b. The peak energies of the bright excitons and combination of bright multiple excitons in the WSe_2_ ML on the Au and Al_2_O_3_ plates were 1.650 and 1.617 eV, respectively. The difference in the peak energy between the dark and bright excitons was 42 meV, which is consistent with a spin‐forbidden dark exciton.^[^ ^59^, ^60^ ^]^ As demonstrated by the increased PL intensity of the WSe_2_ ML on the Au MP edge in Figure 2a, the charge transfer did not substantially affect the PL properties of the WSe_2_ ML on the Au MP edge. Note that the strain‐induced peak shift in the WSe_2_ ML on the MP edges compared to that of the WSe_2_ ML on the plate substrate was not noticeable because the strain was localized by the fairly sharp MP edge (Figure S8, Supporting Information).

The strong emission of dark excitons from the WSe_2_ ML on the Au MP edge at room temperature without an external field can be explained by exciton funneling, as shown in Figure 2e,f. In Figure 2e, X_0_ and X_D0_ denote the bright and dark excitons, respectively. The spin‐forbidden dark excitons in the WSe_2_ ML had an energy difference of ≈40 meV from the spin‐allowed bright excitons.^[^ ^59^ ^]^ The lifetime of dark excitons in the WSe_2_ ML is more than 10^2^ times that of bright excitons, resulting in a relatively longer diffusion or drift distance for dark excitons than that of bright excitons.^[^ ^28^ ^]^ As shown in Figure 2f, the diffusion and emission of bright excitons with a lifetime of ≈2 ps occurred in the WSe_2_ ML on the Al_2_O_3_ plate. In the WSe_2_ MLs on the Al_2_O_3_ and Au MP edges, only dark excitons with lifetimes longer than 10^2^ ps reached the strain‐induced energy potential of the MP edge, and dark exciton emission was observed at the MP edge. Note that for the WSe_2_ ML on the Au MP edge, the dark exciton emission was amplified by the resonance between the dark exciton and SP of Au. Consequently, a strong dark exciton emission was observed in the WSe_2_ ML on the Au MP edge at room temperature without an external field.

### 2.3 Local Field Enhancement for Observing Dark Excitons in the WSe_2_ ML on Au MPs

**Figure 3:**
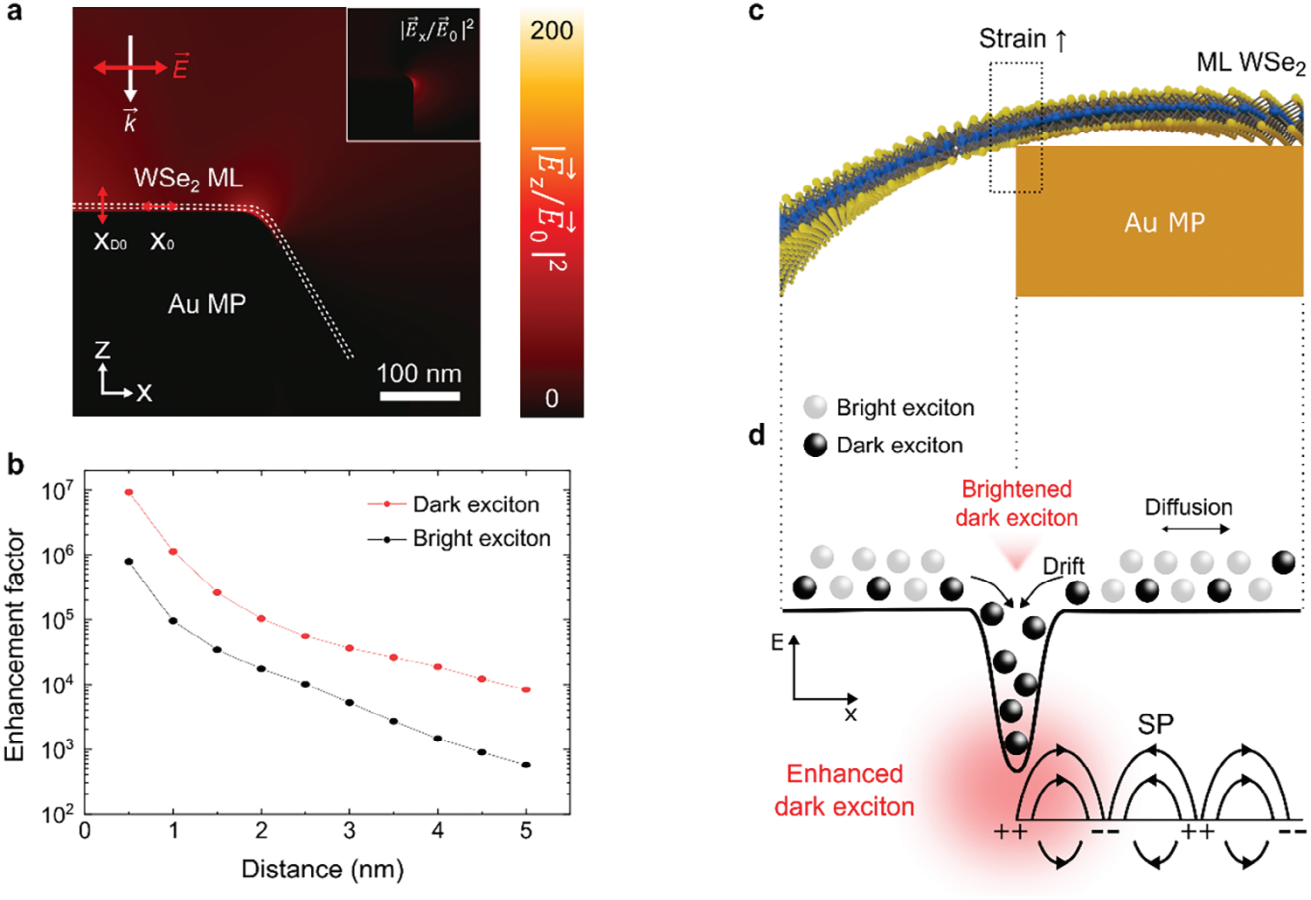
Mechanism of PL enhancement and the observation of dark excitons in the WSe_2_ ML on Au MPs. a) FDTD simulation results exhibiting field enhancement at the edge of the Au MP. b) Plot of the enhancement factor as a function of the gap between the Au MP and WSe_2_ ML. Illustrations of the c) WSe_2_ ML at the edge of the Au MP and d) strain‐induced energy potential. Both bright (white spheres) and dark (black spheres) excitons drift and diffuse to this potential, but only dark excitons with longer lifetimes can reach the energetic minimum.

FDTD simulations were conducted to verify the PL enhancement of dark excitons by localized fields and plasmons. The propagation direction of the incident plane wave was selected to be along the z‐axis, and the electric field was assumed to be polarized along the x‐axis; X_0_ and X_D0_ in **Figure** **3**a represent the bright and dark excitons, respectively. Figure 3a demonstrates that the strong enhancement of the local electric field was explicitly confined to the edge of the Au MP, resulting in an enhanced excitation rate in the localized region. The enhancement factor (EF) of the PL intensity was calculated as follows:

(1) $$\mathrm{EF}={\left(\frac{E}{E_{0}}\right)}^{2}\times F_{P}$$

where *E* is the enhanced electric field at the WSe_2_ ML on the Au MP, and *E_0_* is the initial amplitude of the electric field at the input source. The Purcell factor *F*p was calculated at wavelengths of 751 and 771 nm, considering the bright exciton and dark exciton located at the WSe_2_ ML, respectively. The z‐ and x‐axis components of the enhanced electric‐field intensities were considered separately to calculate the EF of the dark and bright excitons. Because dark excitons are out‐of‐plane oriented whereas bright excitons are in‐plane oriented, two different components of the electric field were considered. The inset presents the electric field intensity enhancement of the bright excitons, which was relatively smaller than that of the dark excitons. Changes in the EF of the dark and bright excitons were plotted as a function of the distance between the WSe_2_ ML and the Au MPs, as shown in Figure 3b. The EF exponentially increased as the distance between the WSe_2_ and Au MPs decreased from 5.0 to 0.6 nm. The PL enhancement of the dark excitons below a gap of 1.0 nm was ≈1000‐fold greater than that at a gap of 5.0 nm. Furthermore, the EF of the dark exciton exhibited a 100‐fold increase compared with that of the bright exciton, which is attributed to the greater electric field enhancement and Purcell factor of the z‐axis‐oriented dark excitons. The WSe_2_ ML can experience a significant tensile strain at the edge of the Au MP, resulting in a reduced distance between the WSe_2_ ML and Au surface that is smaller than the van der Waals gap, leading to a remarkably strong local field enhancement. The relationship between the large local strain of the WSe_2_ ML at the edge of the Au MP and the gap between the WSe2 ML and Au MP must be analyzed to observe the intense dark excitons. Figure 3c presents a cross‐sectional 3D schematic of the WSe_2_ ML deformed under a high strain at the edge of the Au MP, where the local strain of the WSe_2_ ML was maximized and the distance between the WSe_2_ ML and Au MP was correspondingly minimized. Based on these findings, the possible exciton diffusion and drift within a WSe_2_ ML can be depicted along with an energy band diagram. Figure 3d shows a schematic of the exciton diffusion and drift of the WSe_2_ ML at the edge of the Au MP, as well as an energy band diagram. The white and black spheres represent the bright and dark excitons, respectively, and the vertical dashed lines indicate the positions corresponding to the 3D schematic shown in Figure 3c. The energy potential in the WSe_2_ ML was generated by the local strain. Both dark and bright excitons diffuse and drift in the WSe_2_ ML; however, dark excitons with sufficiently long lifetimes can reach this energetic minimum.^[^ ^28^ ^]^ The dark excitons reached the energetic minimum of the local potential and resonated with the Au SP, thereby amplifying the PL intensity. This is owing to the fact that the distance between the WSe_2_ ML and Au surface is significantly small in this local potential region. Consequently, the combination of exciton funneling owing to the local strain of the WSe_2_ ML and the SP resonance of Au enabled the observation of a substantially intense dark exciton emission at room temperature.

### 2.4 Confocal PL Mapping and Spatio‐Spectral Analysis of the WSe_2_ ML on Au MPs

**Figure 4:**
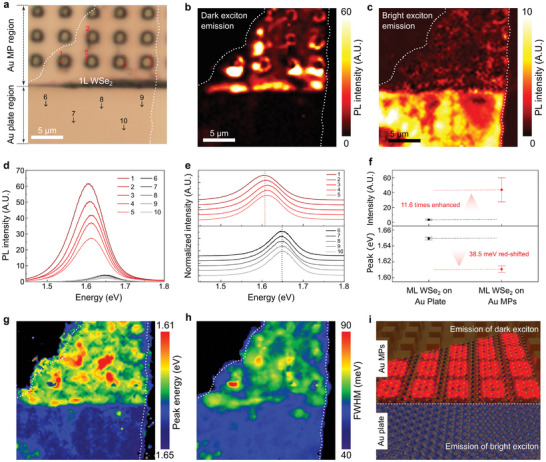
Confocal PL mapping of the WSe_2_ ML on the Au MPs. a) Optical microscopy image of the WSe_2_ ML placed on the Au MPs and Au plate; the white dotted lines indicate the boundary of the WSe_2_ ML. Confocal PL mapping images of the b) dark and c) bright excitons. d) Local PL spectra of the WSe_2_ ML extracted from the representative five points in the Au MP (red) and Au plate (black) regions. e) Intensity normalized PL spectra of the WSe_2_ ML obtained from Au MP and Au plate regions corresponding to the dark (red lines) and bright (black lines) excitons, respectively. f) Comparison of the statistical values of the PL intensity and PL peak energy in the WSe_2_ ML on the Au MP and Au plate regions. 2D distribution of the peak energy g) and FWHM h) of the PL spectra. i) 3D illustration of the dark exciton emission of the proposed device.

To study the spatial distribution of the dark excitons in the WSe_2_ ML on the Au NPs, confocal PL imaging was performed at room temperature. **Figure** **4**a shows an optical photograph of the WSe_2_ ML placed on the Au MPs and the Au plate. The white dotted line marks the edge of the WSe_2_ ML. The five representative points at the Au MP edges (points 1–5) and on the Au plate (points 6–10) are marked with red and black arrows, respectively. Figure 4b,c shows the PL intensity images corresponding to the dark (1.608 eV) and bright (1.648 eV) exciton peaks. The dark exciton PL is strong in the Au MP region, particularly at the edges, which is attributed to the strain‐induced local potential and SP coupling. Note that the height of the Au plate edge is the same as that of the Au MP edge; thus, the dark exciton emission in WSe_2_ ML is stronger at the Au plate edge. Conversely, the PL intensity of the bright excitons in the Au‐plate region was significantly higher than that in the Au MPs region (Figure 4c), indicating that dark exciton emission cannot be observed without a local strain and electric field enhancement. In addition, the confocal PL measurement results with respect to the z‐axis (depth profiling) confirmed that dark exciton emission occurred from the WSe_2_ ML placed on the top surface of the Au MPs (Figure S9, Supporting Information). For a further analysis of the spectroscopic information as well as the PL intensity of the dark exciton of the WSe_2_ ML on the Au MPs, the local PL spectra were extracted at the positions indicated in Figure 4a. The PL peak intensities in the Au MPs regions (red) were 8–18 times higher than the average PL peak intensity in the Au plate region (black). The difference in the peak energy between the two groups was ≈40 meV, with nearly identical peak energies for each group (Figure 4e). The PL peak energies of the WSe_2_ ML on the Au MPs and Au plate were ≈1.610 and ≈1.649 eV, respectively. Figure 4f depicts an interval plot for comparing the PL intensity and PL peak energy of the local PL spectra corresponding to dark and bright excitons, exhibiting ≈11.6 times the intensity and an energy difference of ≈38.5 meV. Note that the strain of WSe_2_ ML on Au MPs is slightly different depending on the location, which leads to the deviation of the peak energy of WSe_2_ ML on Au MPs. To achieve uniform strain, it is essential to create a more uniform structure for the Au MPs and use a more precise wet transfer method. The PL peak energy distribution of the WSe_2_ ML is shown in Figure 4g. The peak energy of the WSe_2_ ML on the Au MPs, where the dark exciton is dominant, was significantly lower than that of the WSe_2_ ML on the Au plate, where only bright excitons were observed. In particular, the PL peak shift of the WSe_2_ ML at the edge of the Au MP was considerably larger, indicating that dark excitons were more clearly observed. The FWHM distribution (Figure 4h) also shows a clear distinction between the Au MP and Au plate regions. As shown in Figure 2, the FWHM increased when the dark excitons were observed owing to the local strain effect.^[^ ^40^, ^61^ ^]^ The resulting increase in the PL of the large‐area 2D crystal with a significantly long lifetime of dark excitons can be used for various applications in light‐emitting devices (Figure 4i).

### 2.5 Angle‐Resolved PL Spectroscopy for Confirmation of the Dark Excitons

**Figure 5:**
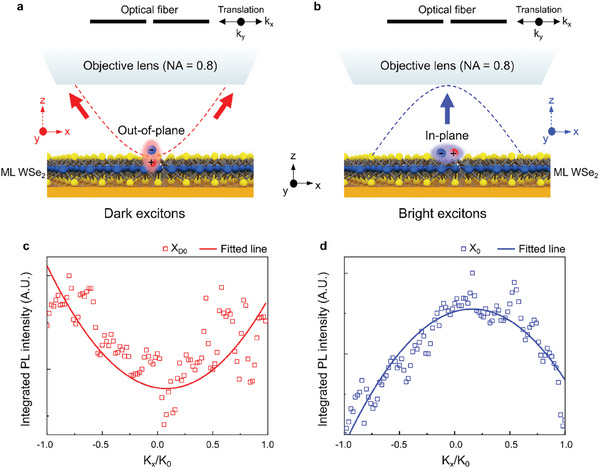
Angle‐resolved PL intensities for the bright and dark excitons. Schematics of the angle‐resolved PL spectroscopy for the a) dark and b) bright excitons in the WSe_2_ ML on the Au MP and Au plate, respectively. A laterally moving optical fiber with a core diameter of 100 µm was used to detect the PL only at a specific detection angle. Integrated PL intensities of the c) dark and d) bright excitons for k_x_‐axis translation.

To further confirm the dark excitons observed in the PL spectrum of the WSe_2_ ML on the Au MPs, angle‐resolved PL spectroscopy was performed. As reported in previous studies, a bright exciton has an in‐plane dipole moment, and a spin‐forbidden dark exciton has an out‐of‐plane dipole moment in the WSe_2_ ML.^[^ ^15^, ^28^, ^42^, ^62^, ^63^ ^]^ Therefore, when the PL spectrum is measured by changing the lateral position of an optical fiber in the Fourier plane, the PL intensities of the bright and dark excitons demonstrate opposite trends, depending on their orthogonal dipole orientation.^[^ ^22^, ^64^ ^]^ The schematics shown in **Figure** **5**a,b present angle‐resolved PL spectroscopy for observing the dark and bright excitons of WSe_2_ ML, respectively. To obtain an angle‐resolved PL spectrum, an optical fiber with a diameter of 100‐µm was placed in the Fourier plane with the 4f‐system and translated in the k_x_‐ and k_y_‐directions. The angle‐resolved PL emissions for the dark and bright excitons were then measured for the WSe_2_ ML on the Au MPs (Figure 5c) and Au plate (Figure 5d), respectively. Because dark excitons have an out‐of‐plane dipole orientation with the in‐plane PL emission, the PL intensity increased as they moved further from the optical axis of the objective lens, as indicated by the red dotted line in Figure 5a. Conversely, bright excitons have an in‐plane dipole orientation, demonstrating a PL emission along the optical axis, as shown in Figure 5b. Hence, the PL intensity of the bright excitons increases as they approach the optical axis of the objective lens. Figure 5c,d show angle‐resolved PL intensity plots of the dark and bright excitons, respectively, which were measured with the optical fiber translated along the k_x_‐axis. The measured PL intensities were normalized and fitted with a nonlinear function to better visualize the angle‐dependent changes in the PL intensity. These results clearly confirm the dark exciton emission of the WSe_2_ ML on the Au MPs.

## 3 Conclusion

In conclusion, the dark exciton emission of the WSe_2_ ML on Au MPs was observed over a large area at room temperature without external magnetic fields. Analyzing the surface morphology demonstrated that the WSe_2_ ML transferred to the Au MP via the wet transfer process was particularly strained at the edge of the Au MPs. Dark exciton emission in the WSe_2_ ML on the Au MPs, induced by the local strain and plasmon enhancement effects, was confirmed via spectral and temporal PL measurements and FDTD simulations. Confocal PL mapping revealed that dark excitons of the WSe_2_ ML were observed over all the Au MPs, particularly at the edge of the Au MP. Furthermore, angle‐resolved PL spectroscopy confirmed the unique dipole‐oriented PL radiation of dark excitons. The fabrication process of the large‐area and bright PL dark exciton devices is simple, inexpensive, and achievable in a short time, indicating a major step toward the practical application of dark excitons in light‐emitting devices, spintronics, photovoltaics, and exciton devices.

## 4 Experimental Section

### Growth of the WSe_2_ ML

The WSe_2_ ML was grown on a SiO_2_/Si substrate using a two‐zone furnace chemical vapor deposition (CVD) system. For the precursors, ammonium metatungstate (AMT, Sigma–Aldrich) solution and pure selenium (Se) pellets (Sigma–Aldrich) were prepared for W and Se, respectively. The Se pellets were loaded into Zone 1 for evaporation in the chamber. For the W precursor, 0.6 g of AMT was dissolved in 30 mL of deionized (DI) water, and the W precursor was spin‐coated onto a cleaned SiO_2_/Si substrate, which was rinsed with organic solvents. The W precursor‐coated substrate was placed at the center of Zone 2. Zone 1 was heated to 380 °C to evaporate the Se, and Zone 2 was heated to 780 °C for 10 min under Ar and H_2_ gases with flow rates of 350 and 5 sccm at atmospheric pressure, respectively. After the growth, the furnace was rapidly cooled to room temperature.

### Fabrication of the WSe_2_ ML on MPs

For the fabrication of the Au MP template, conventional chemical cleaning was applied to the surface of the SiO_2_ substrate. For the shape deposition of the MPs, maskless photolithography (LithoMaskless, Standard Science Inc.) was used with AZ‐GXR‐601(14cp) as the photoresist (P/R), which was developed using AZ‐300 MIF. A 100 nm‐thick gold film was deposited on the prepared P/R MPs using a radio‐frequency sputtering system under a high vacuum for Au MPs. For the Al_2_O_3_ MP template, a 100 nm thick Al_2_O_3_ thin film was deposited on the P/R MP template by atomic layer deposition. For the wet transfer of the WSe_2_ ML on the Au MPs, the CVD‐grown WSe_2_ on a SiO_2_ substrate was transferred using a PMMA‐assisted wet transfer process (Figure S3, Supporting information).

### Characterization

PL measurements were performed at room temperature using a commercial confocal Raman system (LabRam HRevo, HORIBA) with a 532‐nm laser, 100x objective (N.A. = 0.90), and 150 grating (150 groove/500 nm blaze) with a spectral resolution of 0.4 meV. For atomic force microscopy (AFM) measurements, the surface topography of the Au MPs was measured using AFM (XE‐100, Park system) in non‐contact mode at room temperature and atmospheric pressure. For the TRPL measurements, a commercial confocal microscope (Alpha‐300S, WITec Instrument GmbH) equipped with a 100× objective lens (N.A. = 0.9) was used under pulsed excitation at 488 nm (BDL‐488, Becker & Hickl GmbH) with a pulse width of 70 ps, repetition rate of 80 MHz, a high‐speed hybrid detector (HPM‐100‐40, Becker & Hickl GmbH), and a time‐correlated single‐photon counting module (TCSPC, Becker & Hickl GmbH). All the measurements were conducted at room temperature. The surface morphology of WSe_2_ ML on Au MPs was measured by field emission SEM (S‐4800, Hitachi) at an acceleration voltage of 20 kV.

### FDTD Simulation

Finite‐difference time‐domain (FDTD) simulations were conducted using FDTD solutions in Lumerical (ver. 2023R1). The boundary conditions were imposed using a perfectly matched layer method. First, the local electric field E near the edge of the Au substrate was calculated, followed by an evaluation of the enhancement factor as EF = |*E*/*E*_0_|^2^. The excitation wavelength was 532 nm. The incidence is considered to be normal to the Au substrate.

## Conflict of Interest

The authors declare no conflict of interest.

## Supporting information



Supporting Information

## Data Availability

The data that support the findings of this study are available from the corresponding author upon reasonable request.
