# Supplementary material for: Large‐Area Bright Emission of Plasmon‐Coupled Dark Excitons at Room Temperature
Source: Adv Sci (Weinh). 2024 Nov 28;12(3):2411841. doi: 10.1002/advs.202411841 (PMC11744724; doi:10.1002/advs.202411841)
Supplement: Supplementary file 1 — Supporting Information [file ADVS-12-2411841-s001.docx]

Supporting Information

Large-Area Bright Emission of Plasmon-Coupled Dark Excitons at Room Temperature

*Hyun Jeong^1^, Hyeong Chan Suh^1^, Ga Hyun Cho^1^, Huitae Joo^2^, Yeonjeong Koo^2^, Hayoung Ko^3^, Ki Kang Kim^3,4^, Youngbum Kim^3^, Jeongyong Kim^3^, Kyoung-Duck Park^2,^*, and Mun Seok Jeong^1,^**

^1^Department of Physics, Hanyang University, Seoul 04763, Republic of Korea

^2^Department of Physics, Pohang University of Science and Technology (POSTECH), Pohang 37673, Republic of Korea

^3^Department of Energy Science, Sungkyunkwan University, Suwon, 16419, Republic of Korea

^4^Center for Integrated Nanostructure Physics (CINAP), Institute for Basic Science (IBS), Sungkyunkwan University, Suwon, 16419, Republic of Korea

Email: parklab@postech.ac.kr, mjeong@hanyang.ac.kr

KEYWORDS

Dark exciton, monolayer WSe_2_, strain, photoluminescence, surface plasmon

**S1. Optical photograph of periodic Au micro-pillar (MP) arrays**

Photolithography was used to form a square array of periodic MPs over a large area of more than 100 μm × 100 μm. An Au film was then deposited to form periodic Au-MP arrays. Optical microscopy confirmed the formation of a periodic Au MP array over the entire area (Figure S1).


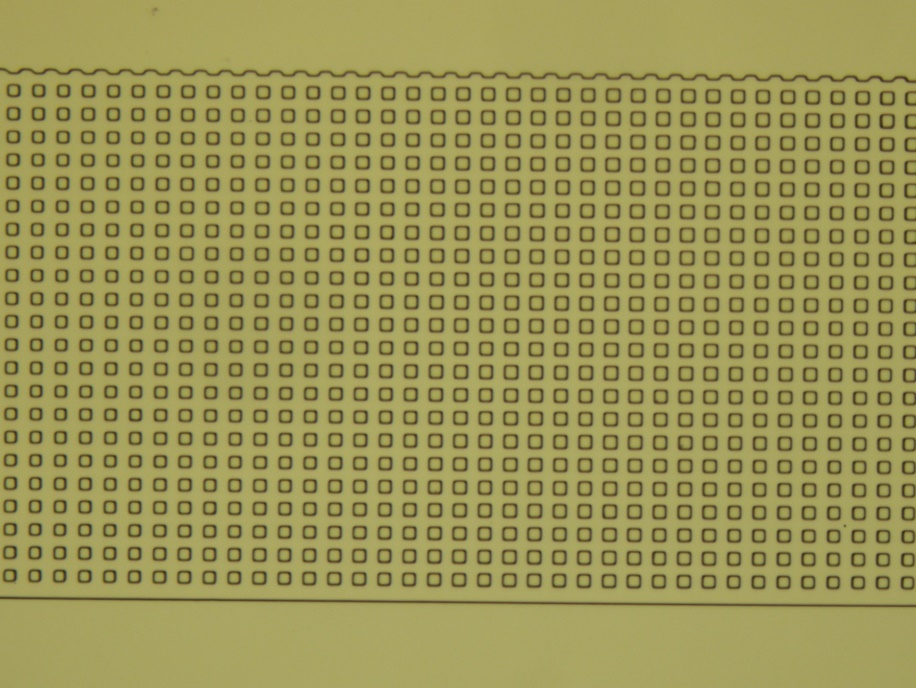


**20 μm**

**Figure S1.** Optical microscopy image of the Au MPs. Uniformly-sized Au MPs were formed over a large area in a square array using photolithography.

**S2. Surface morphology of Au MPs arrays**

The surface morphology of the Au MP array was confirmed using scanning electron microscopy (SEM). The Au MPs were arranged in a square array with a uniform size and spacing, as confirmed by the low-magnification SEM images (Figure S2a). The high-magnification SEM image demonstrates that the Au MP formed with a flat surface and a slightly inclined side (Figure S2b). In particular, the edge of the top surface of the Au MP was significantly abrupt owing to the Au deposition.

**
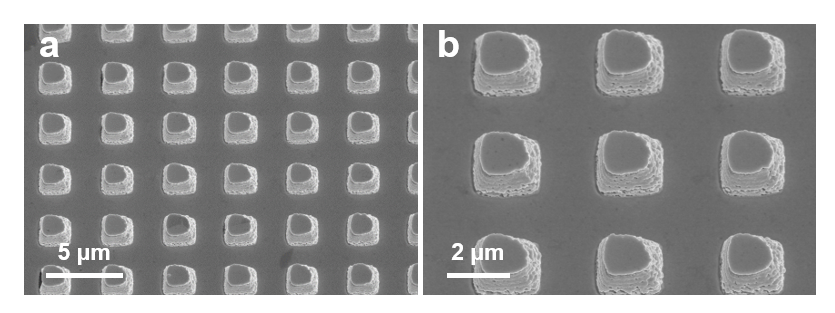
**

**Figure S2.** Perspective SEM image of the Au MPs template. SEM images of the surface of the Au MPs template obtained at a magnification of (a) 5000 and (b)10000 times. The Au MPs are homogeneous in size and spaced at regular distances.

**S3. Wet transfer process of the monolayer (ML) WSe_2_ onto the Au MPs**

A poly(methyl methacrylate) (PMMA)-assisted wet transfer method was used to transfer the WSe_2_ ML onto the Au MPs. The ML of WSe_2_ flakes was grown via chemical vapor deposition (CVD) on a SiO_2_/Si substrate (Figure S3a). PMMA was coated onto the CVD-grown WSe_2_ ML to hold and float the WSe_2_ ML during the subsequent wet transfer process (Figure S3b). The PMMA/WSe_2_ ML on the SiO_2_/Si substrate was immersed in an HF solution diluted with deionized water to etch the SiO_2_ layer (Figure S3c,d). The PMMA/WSe_2_ ML floating on a water-based solution was lifted using the prepared Au MPs template (Figure S3e). Acetone was added dropwise to remove PMMA from the PMMA/WSe_2_ ML (Figure S3f). Finally, transfer of the WSe_2_ ML onto the Au MPs was achieved, as shown in Figure S3g.

**
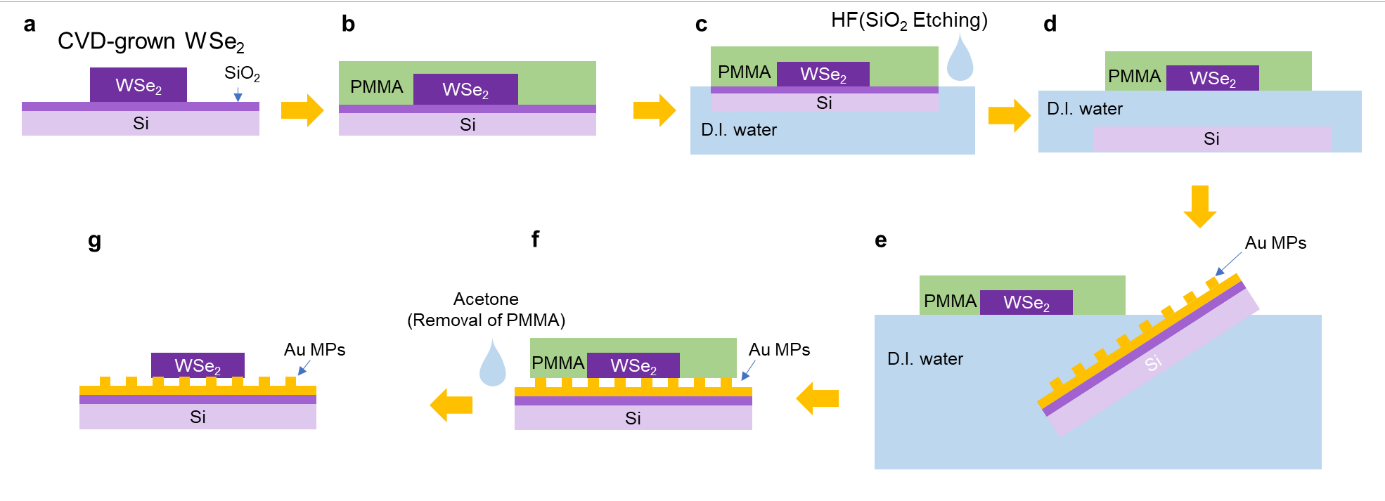
**

**Figure S3.** Wet-transfer process of the WSe_2_ ML from the SiO_2_ substrate to the Au MPs. Cross-sectional schematics of the (a) CVD-grown WSe_2_ ML on 300-nm-thick SiO_2_ on the Si substrate. (b) Poly(methyl methacrylate) (PMMA) coating using a spin coater. (c) Removal of the SiO_2_ layer using the HF solution in DI water. (d) Natural separation of the WSe_2_ ML with PMMA from the Si substrate owing to the removal of the SiO_2_ layer. (e) To transfer the WSe_2_ ML onto an Au MP template, the Au MP template was submerged in DI water to deposit the WSe_2_ ML with PMMA. Schematics of the (f) WSe_2_ ML with PMMA placed on the Au MPs and the (g) WSe_2_ ML on the Au MPs after removal of PMMA.

**S4. Topological analysis of WSe_2_ ML**

The flake shape and layer thickness of the CVD-grown WSe_2_ ML were verified by optical microscopy and atomic force microscopy (AFM). Figure S4a presents an optical image of a WSe_2_ ML flake, which is triangular at the edge. Figure S4b presents the surface topography of a WSe_2_ ML on a SiO_2_ substrate. The height profile along the white dotted line is indicated by the topography marked as a white solid line. The measured thickness of the WSe_2_ ML was 0.7 nm, which is consistent with the thickness of a typical WSe_2_ ML.

**
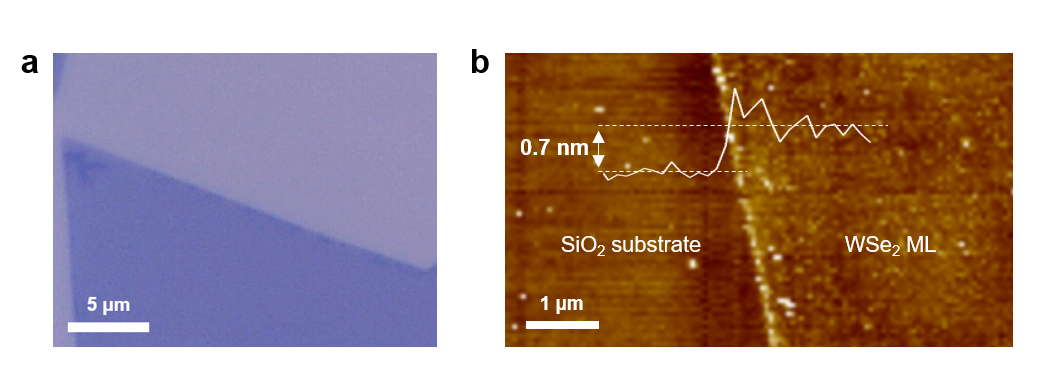
**

**Figure S4.** Characterization of the WSe_2_ ML structure. (a) Optical microscopy image of the CVD-grown WSe_2_ ML on a SiO_2_ substrate. (b) AFM image of the WSe_2_ ML on a SiO_2_ substrate; the inset graph presents the height profile along the white dotted line. The height of the WSe_2_ ML was measured to be 0.7 nm, which is equivalent to the WSe_2_ monolayer.

**S5. WSe_2_ ML on Au MPs: Strong local strain and crystal deformation**

Figure S5a presents the SEM image of a specific area of the WSe_2_ ML on the Au MPs. The WSe_2_ ML broken between the Au MPs demonstrates that the wet transfer process subjected the WSe_2_ ML to high strain at the edges of the Au MPs, which was responsible for the dark excitons observed at room temperature. Figure S5b depicts a 3D schematic of the deformed WSe_2_ ML at the edge of the Au MP, which was strained in all directions. Owing to the pulling force from all directions of the WSe_2_ ML at the edge of the Au MP, the strain of the WSe_2_ ML was maximized at the edge, which was the basis for the observation of the dark excitons.

**
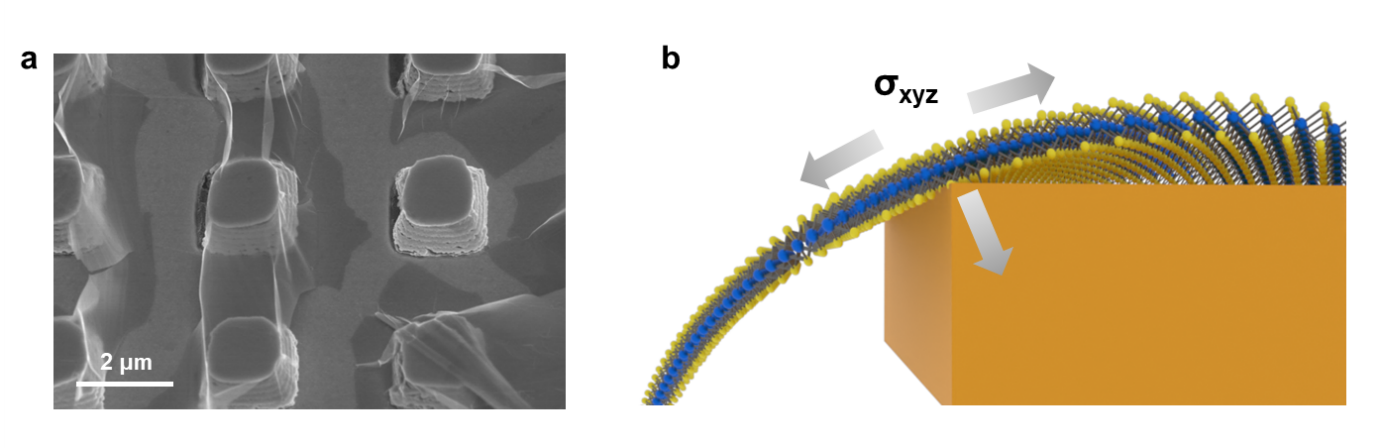
**

**Figure S5.** Strong omnidirectional strain in the WSe_2_ ML at the Au MP edge. (a) SEM image of the WSe_2_ ML broken under a strong strain between the Au MPs. (b) 3D schematic of the WSe_2_ ML subjected to a high strain in all directions at the Au MP edge, deforming its shape.

**S6. Strain of the WSe_2_ ML at the edge of the Au MP**

The strain of WSe_2_ ML at the Au MP edge was calculated based on the morphological characteristics of the Au MP edge. As shown in the SEM image in Figure S6a, a discontinuous Au film is formed during the sputtering of the Au film on the P/R MP. In particular, a thin layer of Au film is formed on the top surface of the MP. In the magnified SEM image of the inclined plane including the top surface, a thin Au film is formed on the top surface and the edge shows a considerably small radius. (Figure S6b) The radius of the Au MP edge is 30 - 50 nm.

Based on these values, the strain of WSe_2_ ML at the Au MP edge was calculated according to the following equation.^[1]^

$$\varepsilon=\frac{\tau}{2R}\times100 (\%)$$

where ε is the strain, τ is the thickness of the sample, and R is the radius of curvature. (Figure S6c) The strain is calculated to be 0.70% to 1.17% at a thickness of 0.7 nm for the WSe_2_ ML and a radius of 30 to 50 nm for the Au MP edge.

**
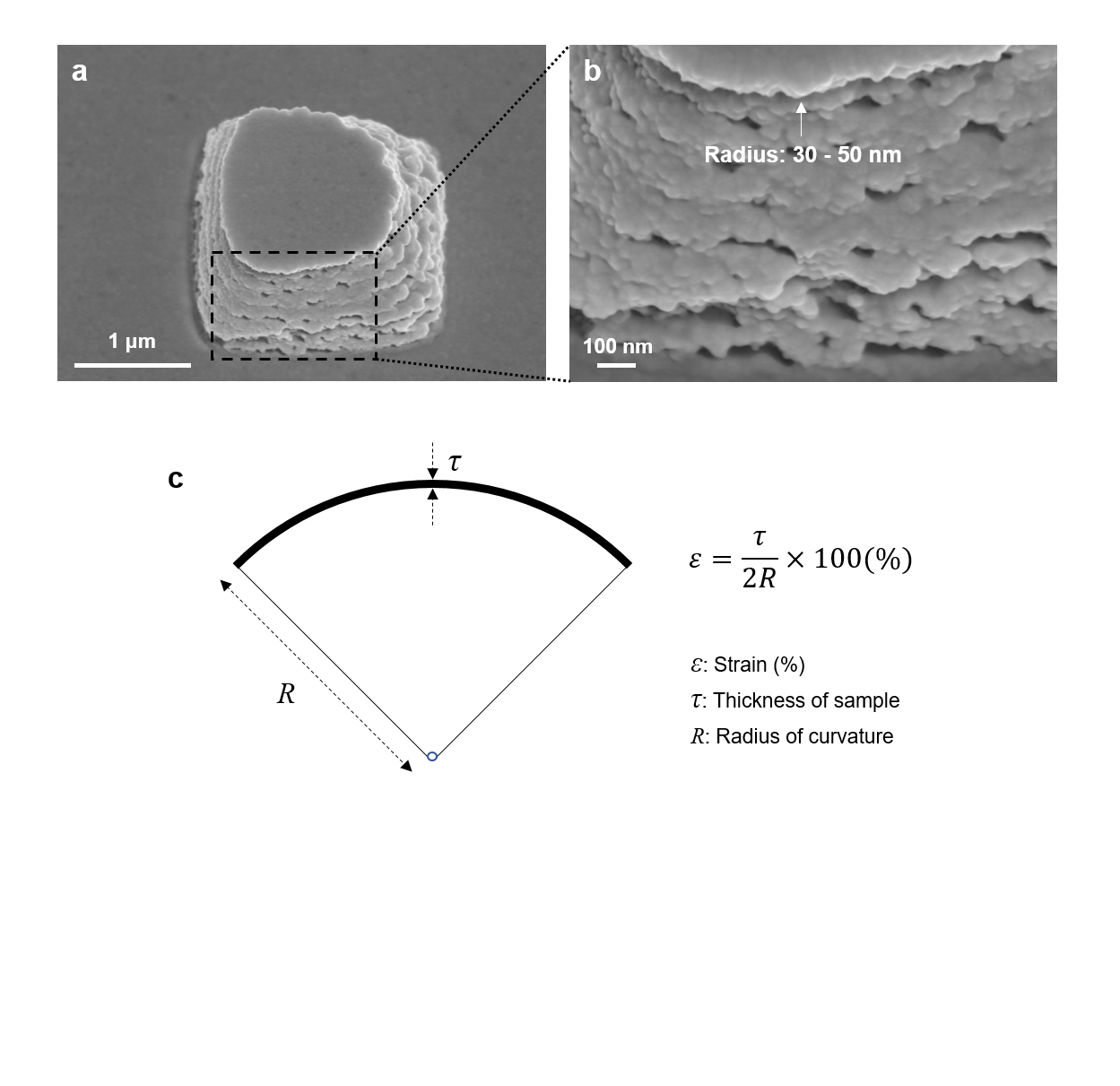
**

**Figure S6.** SEM images of single Au MPs and strain calculations. (a) Low and (b) high magnification SEM images of single Au MPs. (c) Strain calculation of WSe_2_ ML at the edge of the Au MP.

**S7. Comparison of the PL lifetime of the WSe_2_ ML depending on the substrate**

The average PL lifetimes of the WSe_2_ ML on the Au plate, Au MPs, and Al_2_O_3_ MPs were 104, 161, and 305 ps, respectively. Notably, the TRPL used in this study had a temporal resolution of 100 ps. Therefore, the lifetime of 104 ps for the WSe_2_ ML on the Au plate was attributed to the resolution limit of the instrument and not the actual lifetime of the WSe_2_ ML. Regardless, the lifetimes of the WSe_2_ ML on the Au and Al_2_O_3_ MPs corresponding to the dark excitons were significantly higher than those of the WSe_2_ ML on Au plates corresponding to the bright excitons. The actual lifetime of the WSe_2_ ML is a few picoseconds, as reported in literature.

**
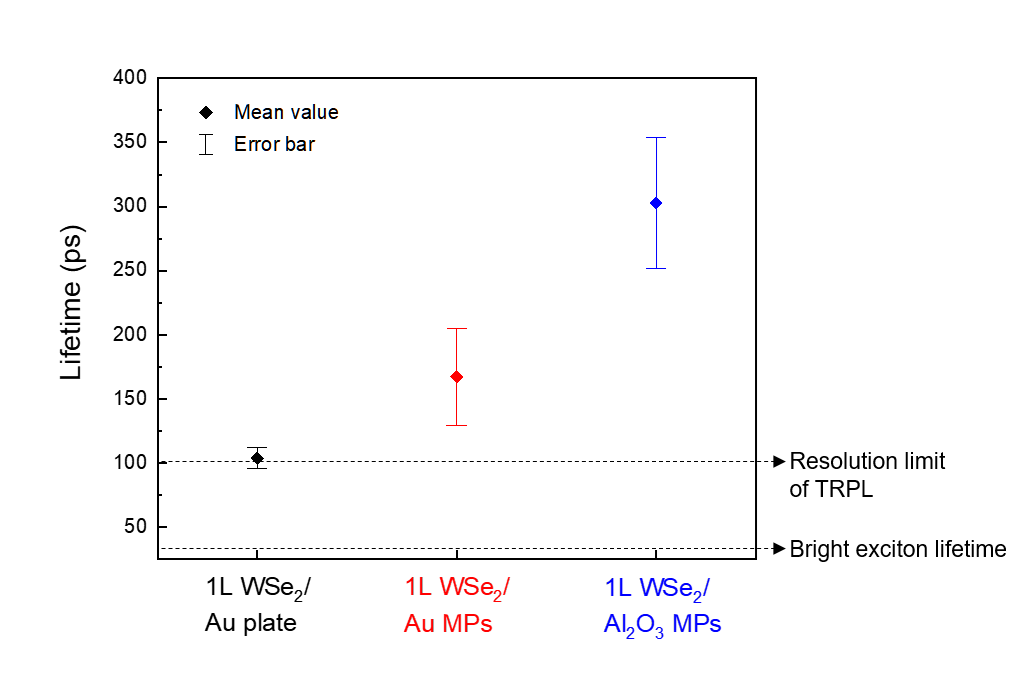
**

**Figure S7.** PL lifetime of the WSe_2_ ML on the Au plate, Au MPs, and Al_2_O_3_ MPs.

**S8. Localized strain region of WSe_2_ ML along MP edge.**

It is well known that the bright exciton peak of WSe_2_ ML should be red-shifted by strain. However, in this study, the bright exciton peak on WSe_2_ ML on the MP edge did not show a noticeable change in peak position compared to WSe_2_ ML on the flat substrate. This is attributed to the highly localized strain region. Figure S8a presents a highly localized region subjected to a strong strain at the WSe_2_ ML on the Au MP edge. As depicted in the 3D image, the strain region of the WSe_2_ ML along the MP edge is highly localized. As shown in Figure S8b, considering the focused beam diameter of 800 nm and the width of the area where WSe_2_ ML is strained at the MP edge (60 nm), the laser spot area occupies 90.4%, while the local strain area is only 9.6%. Therefore, the strain region of WSe_2_ ML on MPs is highly localized, resulting in the peak position appearing almost unchanged in the PL spectrum.


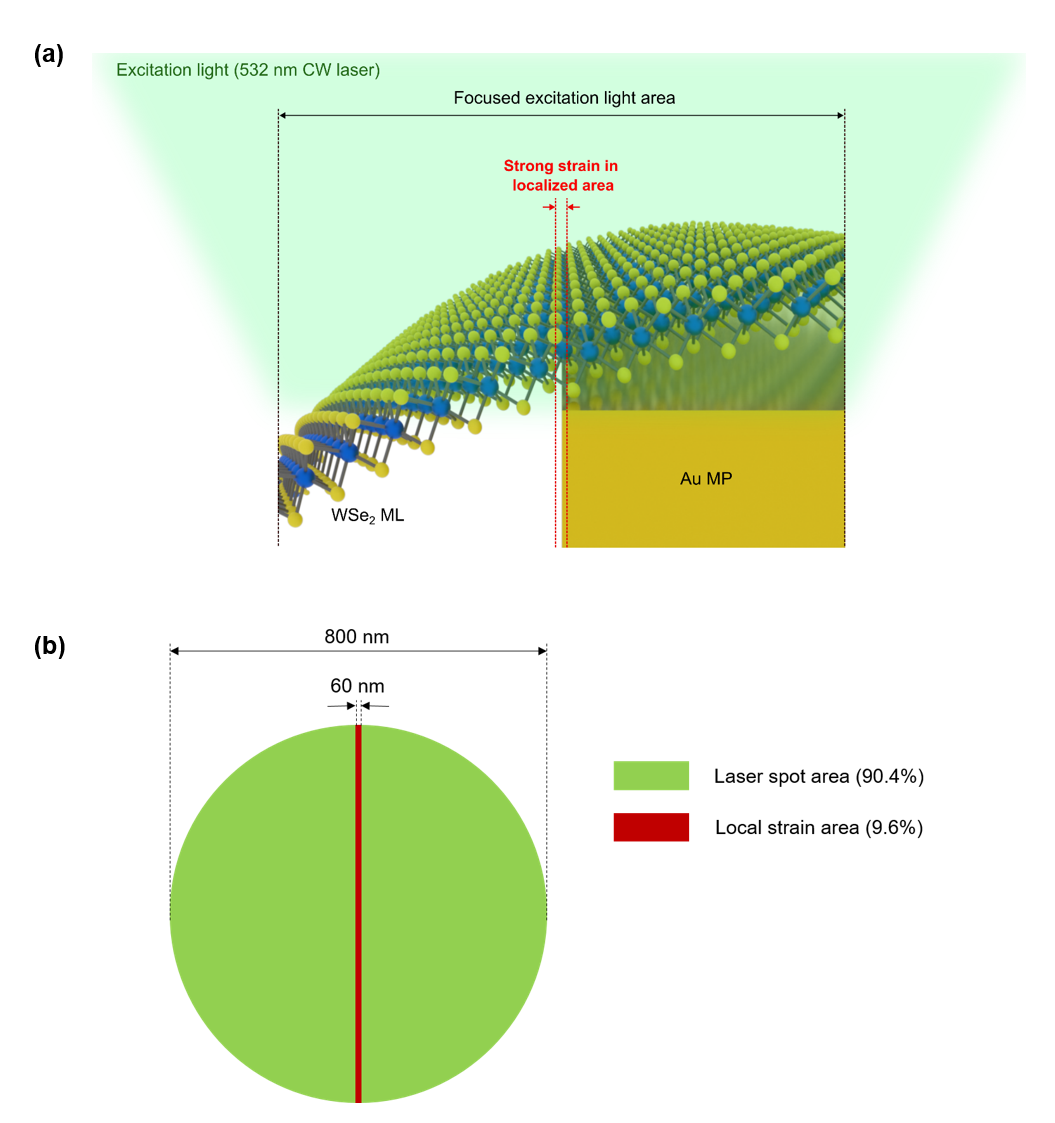


**Figure S8.** Localized strain region of WSe_2_ ML along MP edge. (a) 3D schematic of the WSe_2_ ML on the Au MP edge with the excitation light. (b) Proportion of local strain area within the laser spot.

**S9. Confocal PL mapping along z-axis**

Confocal PL spectroscopy was performed along the focal plane to verify the region where the dark exciton of the WSe_2_ ML on the Au MP was observed. Figure S9a shows the focal plane dependence of the confocal PL spectra of the WSe_2_ ML on the Au MP. Plane 2 is the focal plane of the top surface of the Au-MP. Planes 1 and 3 were the focal planes 600 nm above and 600 nm below the top surface of the Au MP, respectively. The local PL spectra, marked by the black, red, and blue solid lines, were extracted from planes 1, 2, and 3, respectively (Figure S9b). At the top surface of the Au MP, the PL intensity of WSe_2_ ML is the strongest and, at the same time, the dark exciton component is the most dominant. However, at the focal plane above or below the top surface of the Au MP, the PL intensity of the WSe_2_ ML was relatively low and the dark exciton component was relatively small. Because the dark exciton of the WSe_2_ ML is observed on the top surface of the Au MP, where the strain is the strongest, this implies that the dark exciton of the WSe_2_ ML on the Au MPs is closely related to the strain.

**
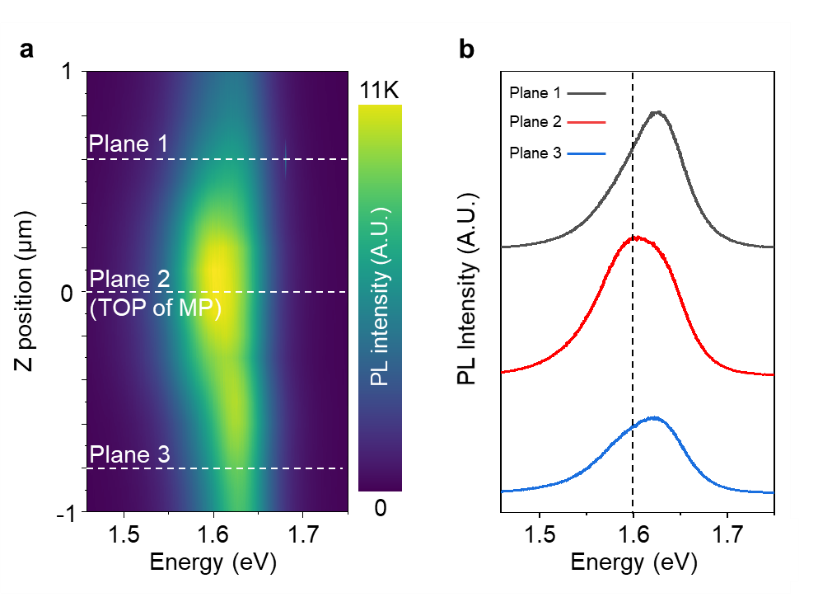
**

**Figure S9.** Confocal PL spectroscopy of WSe_2_ ML on Au MP according to focal plane at the edge region of Au MP. (a) Spectral intensity versus focal plane of confocal PL spectroscopy. The PL intensity is strongest in focal plane 2, which is the top surface of Au MP, and the PL peak position is more redshifted at this focal plane. (b) Local PL spectra extracted from focal plane 1 (black), 2 (red), and 3 (blue). The PL spectrum extracted from focal plane 2 contains the highest dark exciton component, which supports the observation of dark excitons at Au MP.

References

[1] A. C. Ugural, *Mechanics of Materials*, Wiley, **2007**.
